# Use of the informational spectrum methodology for rapid biological analysis of the novel coronavirus 2019-nCoV: prediction of potential receptor, natural reservoir, tropism and therapeutic/vaccine target

**DOI:** 10.12688/f1000research.22149.4

**Published:** 2021-01-06

**Authors:** Veljko Veljkovic, Júlia Vergara-Alert, Joaquim Segalés, Slobodan Paessler

**Affiliations:** 1 Biomed Protection, Galveston, TX, 77550, USA; 2 IRTA, Centre de Recerca en Sanitat Animal (CReSA, IRTA-UAB), Campus de la Universitat Autònoma de Barcelona, Barcelona, 08193 Bellaterra, Spain; 3 Departament de Sanitat i Anatomia Animals, Universitat Autònoma de Barcelona (UAB), Barcelona, 08193 Bellaterra, Spain; 4 UAB, Centre de Recerca en Sanitat Animal (CReSA, IRTA-UAB), Campus de la Universitat Autònoma de Barcelona, Barcelona, 08193 Bellaterra, Spain; 5 Department of Pathology, Galveston National Laboratory, University of Texas Medical Branch, Galveston, TX, 77555, USA

**Keywords:** 2019-nCoV, Wuhan coronavirus, SARS, MERS

## Abstract

A novel coronavirus recently identified in Wuhan, China (SARS-CoV-2) has expanded the number of highly pathogenic coronaviruses affecting humans. The SARS-CoV-2 represents a potential epidemic or pandemic threat, which requires a quick response for preparedness against this infection. The present report uses the informational spectrum methodology to identify the possible origin and natural host of the new virus, as well as putative therapeutic and vaccine targets. The performed *in silico* analysis indicates that the newly emerging SARS-CoV-2 is closely related to severe acute respiratory syndrome (SARS)-CoV and, to a lesser degree, Middle East respiratory syndrome (MERS)-CoV. Moreover, the well-known SARS-CoV receptor (ACE2) might be a putative receptor for the novel virus as well. Actin protein was also suggested as a host factor that participates in cell entry and pathogenesis of SARS-CoV-2; therefore, drugs modulating biological activity of this protein (e.g. ibuprofen) were suggested as potential candidates for treatment of this viral infection. Additional results indicated that civets and poultry are potential candidates for the natural reservoir of the SARS-CoV-2, and that domain 288-330 of S1 protein from the SARS-CoV-2 represents promising therapeutic and/or vaccine target.

## Introduction

Fears are mounting worldwide over the cross-border spread of the new coronavirus (denoted as SARS-CoV-2) that originated in Wuhan, the largest city in central China, after its spread to many countries around the world. The newly emerging pathogen belongs to the same virus family as the deadly severe acute respiratory syndrome and Middle East respiratory syndrome coronaviruses (SARS-CoV and MERS-CoV, respectively). The World Health Organization (WHO) has recently published surveillance recommendations for a possible “large epidemic or even pandemic” (pandemic declared on March 11 ^th^, 2020) of the novel coronavirus and it has issued guidelines for hospitals across the world. However, many questions about SARS-CoV-2 remain unanswered: (i) what is the origin and/or natural reservoir of the virus? (ii) is it easily transmitted from human to human? and (iii) what are the potential diagnostic, therapeutic and vaccine targets? Currently, only nucleotide sequences of eight human SARS-CoV-2 isolates are available without any additional information about biological properties of the virus, beyond the morphological confirmation of the virion using electronic microscopy. This is likely not enough information to answer the important abovementioned questions.

The informational spectrum method (ISM), a virtual spectroscopy method for analysis of proteins, is based on the fundamental electronic properties of amino acids and requires only nucleotide sequence availability to investigate proteins ^1^. For this reason, ISM was previously used for analysis of novel viruses for which little or no information were available ^2– 5^. Here, the SARS-CoV-2 was analyzed with ISM to identify its possible origin and natural host, as well as putative therapeutic and vaccine targets.

## Methods

### Sequences

The S1 surface protein sequences from the first 8 human SARS-CoV-2, deposited in the publicly available GISAID database (assessed on January 19, 2020), were analyzed by ISM. The studied sequences were BetaCoV/Wuhan/IVDC-HB-04/2020, BetaCoV/Wuhan/IVDC-HB-01/2019, BetaCoV/Wuhan/IVDC-HB-05/2019, BetaCoV/Wuhan/IPBCAMS-WH-01/2019, BetaCoV/Wuhan/WIV04/2019, BetaCoV/Wuhan-Hu-1/2019, BetaCoV/Nonthaburi/61/2020, and BetaCoV/Nonthaburi/74/2020.

In the phylogenetic analysis, different amino acid sequences of other coronaviruses were also included: (i) S1 proteins from the following viruses: AVP78042, AVPvp78031, AY304486, AY559093, JX163927, YN2018B, KY417146, used already by other authors in the study of the phylogenetic relationship between SARS-CoV-2 and nearest bat and SARS-like CoVs ( GISAID database); and (ii) S1 proteins from three first isolated human MERS-CoV: AGG22542, AFS88936, AFY13307, deposited in the GISAID database

### The ISM

Detailed description of the sequence analysis based on ISM has been published elsewhere ^2^. According to this approach, sequences (protein or DNA) are transformed into signals by assignment of numerical values of each element (amino acid or nucleotide). These values correspond to electron-ion interaction potential ^6^, determining electronic properties of amino acid/nucleotides, which are essential for their intermolecular interactions. The signal obtained is then decomposed in a periodical function by the Fourier transformation. The result is a series of frequencies and their amplitudes. The obtained frequencies correspond to the distribution of structural motifs (primary structure) with defined physico-chemical characteristics responsible for the biological function of the putative protein corresponding to the analyzed sequence. When comparing proteins that share same biological or biochemical function, the technique allows detection of code/frequency pairs that are specific for their common biological properties. The method is insensitive to the location of the motifs and, therefore, does not require previous alignment of the sequences. In addition, this is the only method that allows immediate functional analysis.

### Phylogenetic analysis

The phylogenetic tree of S1 proteins from coronaviruses was generated with the ISM-based phylogenetic algorithm ISTREE, previously described in detail elsewhere ^7^. In the presented analysis, we calculated the distance matrix with the amplitude on the frequency F(0.257) as the distance measure between sequences.

## Results and discussion

**Figure 1.**
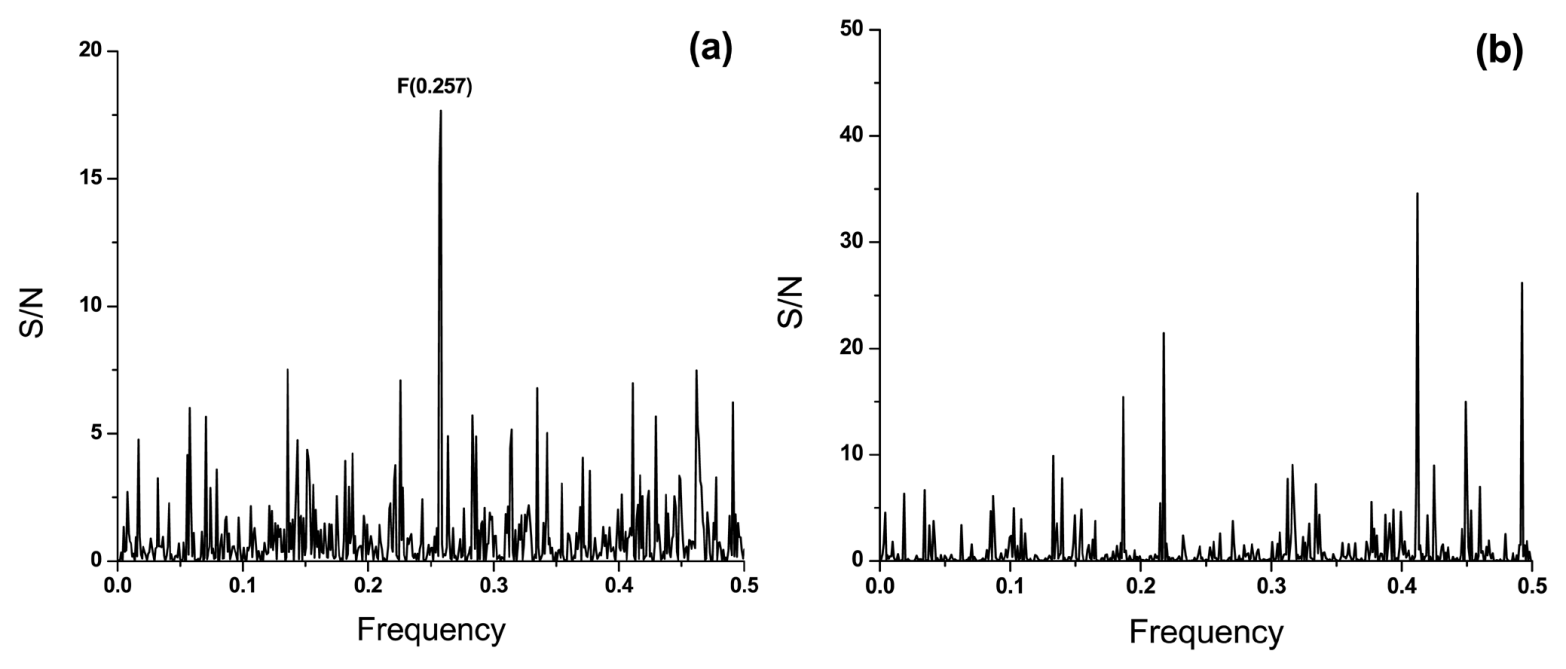
Cross-spectrum (CS) of S1 proteins. ( **a**) CS of S1 from SARS-CoV, MERS-CoV and SARS-CoV-2; ( **b**) CS of Bat SARS-like CoV and SARS-CoV-2. The abscissa represents the frequencies from the Fourier transform of the sequence of electron-ion interaction potential corresponding to the amino-acid sequence of proteins. The lowest frequency is 0.0 and the highest is 0.5. The ordinate represents the signal-to-noise ratio (the ratio between signal intensity at one particular IS frequency and the main value of the whole spectrum, S/N).

In order to compare informational similarity between SARS-CoV-2, SARS-CoV, MERS-CoV and Bat SARS-like CoV, the cross-spectra (CS) of S1 proteins from these viruses were calculated. Figure 1a shows the CS of SARS-CoV-2, SARS-CoV and MERS-CoV. These CS contain only one dominant peak corresponding to the frequency F(0.257). Figure 1b displays the CS of S1 proteins from SARS-CoV-2 and Bat SARS-like CoV. Amplitudes in these latter CS are significantly lower than in those CS presented in Figure 1a. These results show that (i) S1 proteins from SARS-CoV-2, SARS-CoV, MERS-CoV and Bat SARS-like CoV encode common information, which is represented with the frequency F(0.257), and (ii) S1 proteins from SARS-CoV-2 are remarkable more informationally similar with S1 from SARS-CoV and MERS-CoV than with S1 from Bat SARS-like CoV. This suggests that biological properties of SARS-CoV-2 are apparently more similar to SARS-CoV and MERS-CoV than to Bat SARS-like CoV.

**Figure 2.**
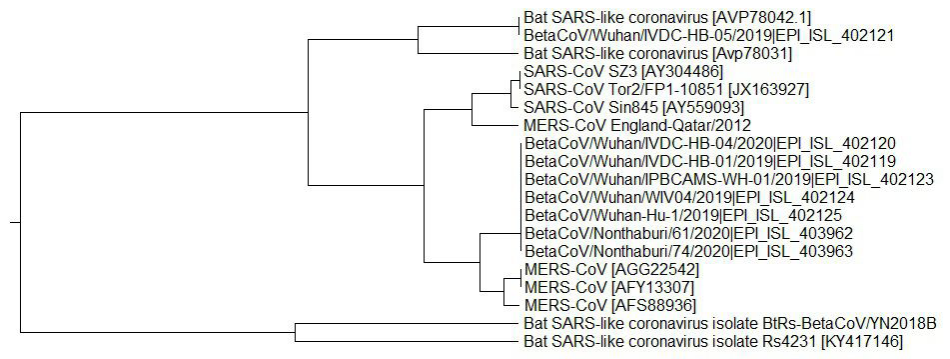
Informational spectrum method-based phylogenetic tree for S1 proteins from SARS-CoV, MERS-CoV, Bat SARS-like CoV and SARS-CoV-2. The frequency F(0.257) as the distance measure was used.

To confirm this conclusion, the ISM-base phylogenetic tree for S1 proteins was calculated ( Figure 2). In this calculation the amplitude on the frequency F(0.257) was used as the distance measure. As observed in Figure 2, all analyzed SARS-CoV-2 S1 amino acid sequences are grouped with SARS-CoV and MERS-CoV and separated from Bat SARS-like CoV. This indicates that SARS-CoV-2 are more phylogenetically similar to SARS-CoV and MERS-CoV than to Bat SARS-like CoV. This result differs from those obtained with the homology-based phylogenetic analysis, which showed that SARS-CoV-2 are closely related to Bat SARS-like CoV ( https://platform.gisaid.org/epi3/frontend#lightbox1296857287).

**Figure 3.**
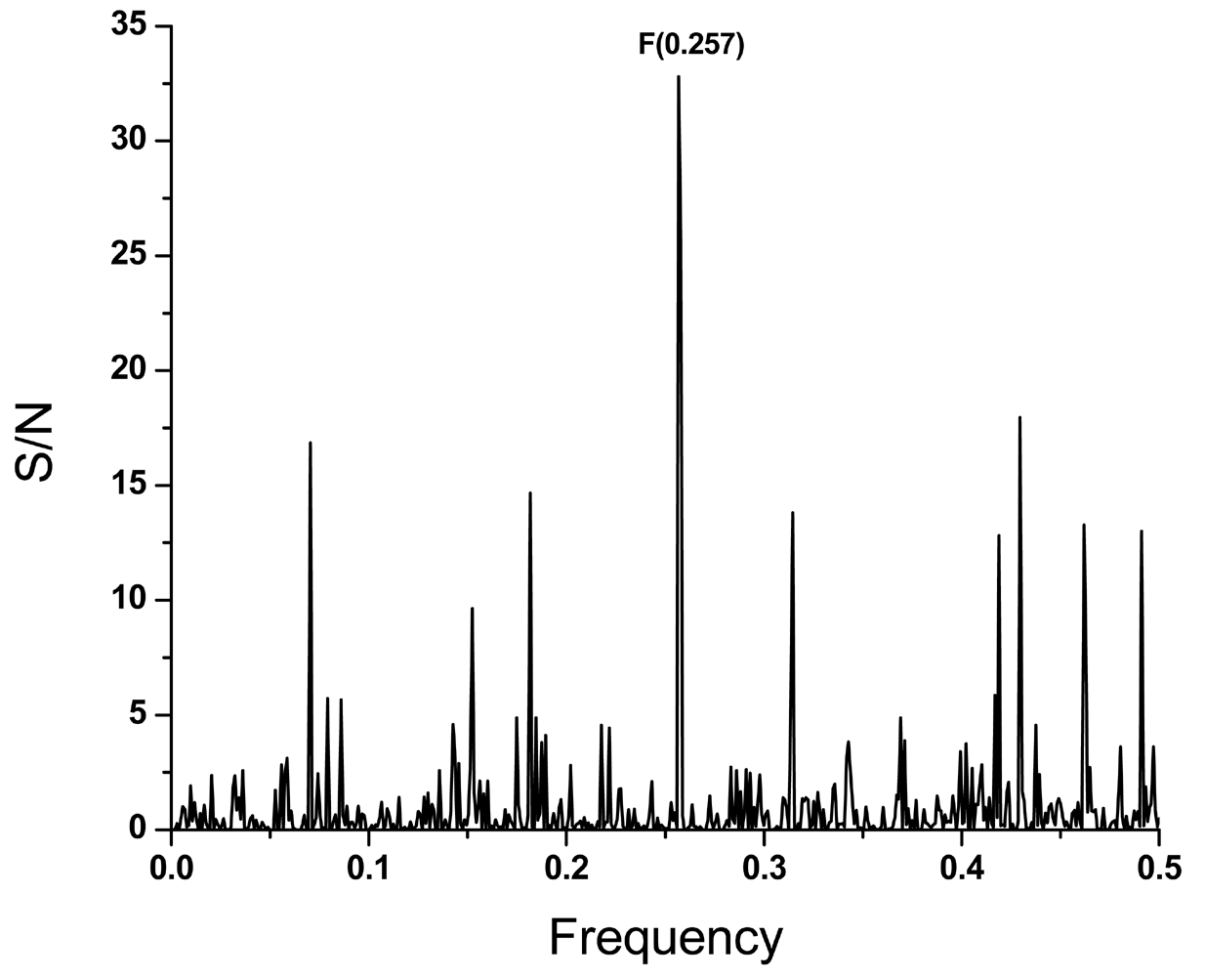
Cross-spectrum of ACE2 and S1 proteins from SARS-CoV and MERS-CoV. The abscissa and the ordinate are as described in Figure 1.

It has been previously shown that the dominant frequency in the informational spectrum of viral envelope proteins corresponds to interaction between the virus and its receptor ^2, 3, 7, 8^. The ISM analysis showed that the frequency component F(0.257) is present in the CS of S1 SARS-CoV and its receptor angiotensin converting enzyme 2 (ACE2) ^9^, but not in the CS of S1 MERS-CoV and its main receptor dipeptidyl peptidase 4 (DPP4) ^10^. Of note is that both receptors ACE2 and DPP4 are expressed in airway epithelia. Presence of F(0.257) in the informational spectrum of MERS-CoV ( Figure 1) suggests also possible interaction between this virus and the ACE2. The dominant peak on the frequency F(0.257) in the CS of S1 from SARS-CoV and MERS-CoV and ACE2 supports this possibility ( Figure 3), although this has not been formally proved for MERS-CoV ^11^.

As it is shown in Figure 1a, the frequency F(0.257) is also present in the informational spectrum of the SARS-CoV-2, suggesting that ACE2 might be the receptor for this novel coronavirus too. This prediction was subsequently confirmed by functional studies *in vitro* ^12^. Calculation of the CS for S1 protein from the SARS-CoV-2 and all ACE2 sequences available at the UniProt database revealed that the highest amplitudes on the frequency F(0.257) correspond to ACE2 from civet and chicken. This result indicates that these species can be included as potential candidates for the natural reservoir of the SARS-CoV-2. However, it is possible that SARS-CoV-2 viruses use very different receptors in the natural host(s) and not only the ACE2 as it is the putative case in humans. An experimental study performed on chicken, however, indicated lack of susceptibility of this species to the novel virus ^13^; civets so far have not been tested, but the indicated study confirmed susceptibility of domestic cat to SARS-CoV-2.

**Figure 4.**
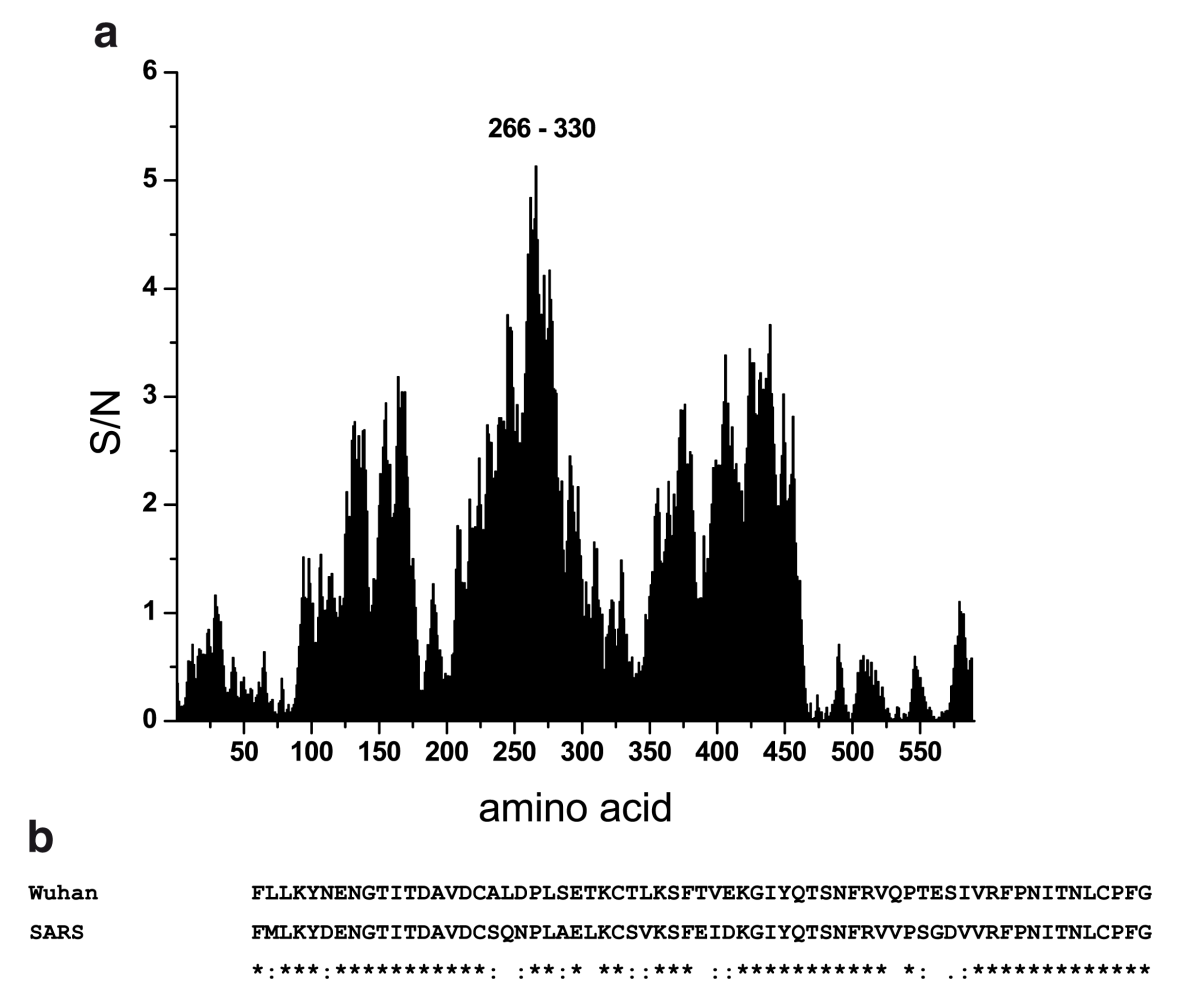
Domain of S1 protein which is important for SARS-CoV-2/ACE2 interaction. ( **a**) Mapping of the domain of S1 protein from SARS-CoV-2 (BetaCoV/Wuhan/IVDC-HB-01/2019) which gives the dominant contribution to the information represented with the frequency F(0.257). ( **b**) Sequence homology between domains of S1 proteins from SARS-CoV and SARS-CoV-2 with essential contribution to the information corresponding to the frequency F(0.257).

Finally, the S1 amino acid sequence from the SARS-CoV-2 was scanned to look for the domain that gives the highest contribution to the information represented by the frequency F(0.257) ( Figure 4a). This analysis revealed domain 266–330 (numbering concerns the maturated protein) is essential for interaction of SARS-CoV-2 with ACE2. Of note is the striking homology between these domains of S1 proteins from SARS-CoV-2 and SARS-CoV, but not from MERS-CoV for which ACE2 is not the main receptor ( Figure 4b).

**Figure 5.**
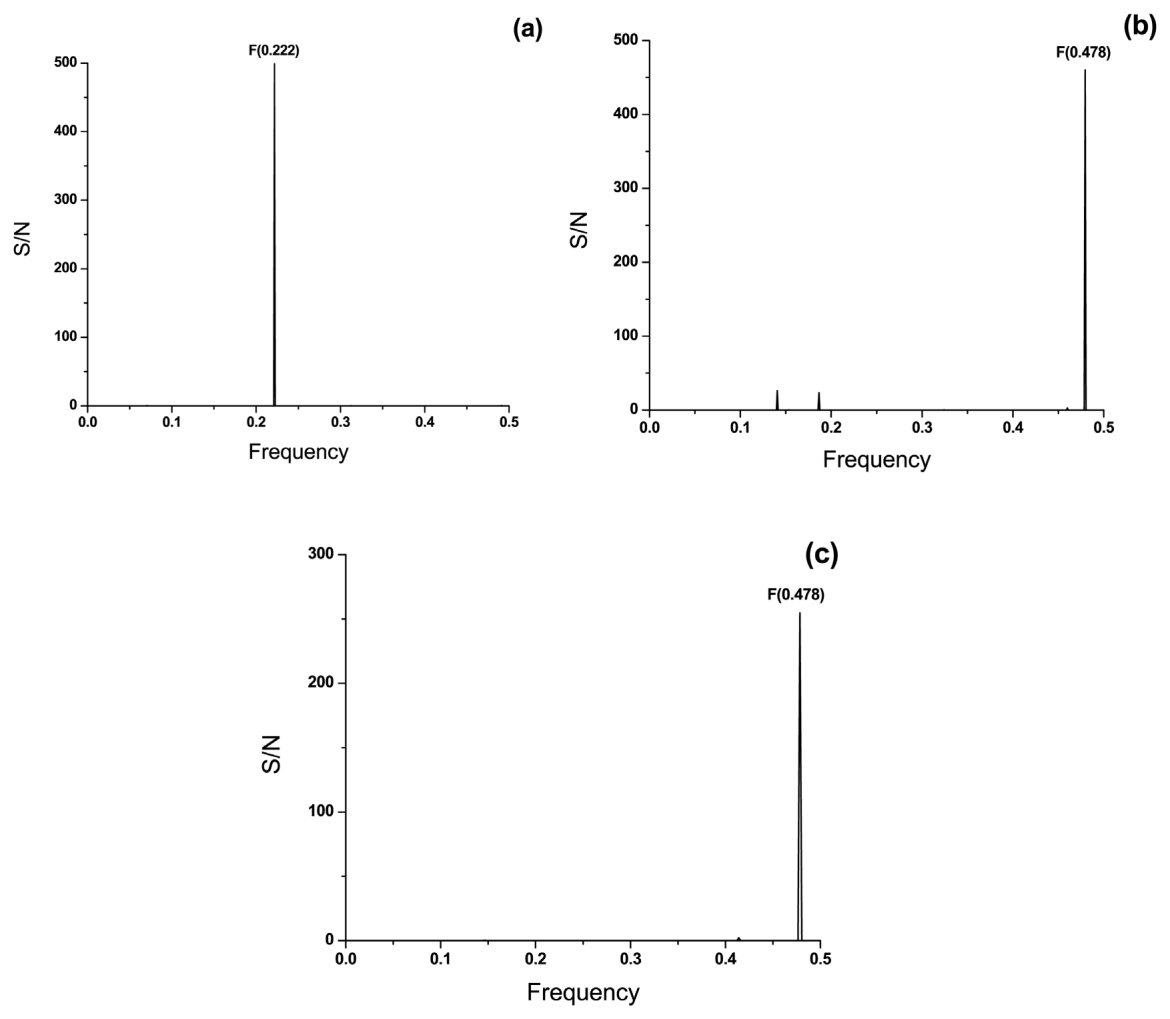
CS of S1 proteins from SARS-CoV and SARS-CoV-2 and actin proteins. ( **a**) CS of S1 proteins from human SARS-CoV; ( **b**) CS of S1 proteins from SARS-CoV-2; ( **c**) CS of mammalian actin proteins. The abscissa and the ordinate are as described in Figure 1.

Further, S1 spike proteins from SARS-CoV ( Table 1) and SARS-CoV-2 ( Table 2) were compared. The CS of S1 proteins from SARS-CoV ( Figure 5a) and SARS-CoV-2 ( Figure 5b) were assessed. Principal information encoded in S1 proteins from SARS-CoV and SARS-CoV-2 is represented with two different frequencies F(0.222) and F(0.478), respectively. This result indicates some potential difference(s) in the virus-host interaction of these two viruses although they apparently use the same receptor ACE2.

**Table 1.** S1 proteins from SARS_CoV ( uniprot.org).

SPIKE_CVHSA Spike glycoprotein OS=Human SARS coronavirus
Q19QX0_CVHSA Spike glycoprotein OS=Human SARS coronavirus
A7J8L4_CVHSA Spike glycoprotein OS=Human SARS coronavirus
J9SFL2_CVHSA Spike glycoprotein OS=Human SARS coronavirus
J9TDZ0_CVHSA Spike glycoprotein OS=Human SARS coronavirus
Q202F4_CVHSA Spike glycoprotein OS=Human SARS coronavirus
Q202E5_CVHSA Spike glycoprotein OS=Human SARS coronavirus
Q202E9_CVHSA Spike glycoprotein OS=Human SARS coronavirus
Q202F5_CVHSA Spike glycoprotein OS=Human SARS coronavirus
Q202E6_CVHSA Spike glycoprotein OS=Human SARS coronavirus
Q202H5_CVHSA Spike glycoprotein OS=Human SARS coronavirus
Q202F2_CVHSA Spike glycoprotein OS=Human SARS coronavirus
Q202F9_CVHSA Spike glycoprotein OS=Human SARS coronavirus
Q202G8_CVHSA Spike glycoprotein OS=Human SARS coronavirus
Q202G3_CVHSA Spike glycoprotein OS=Human SARS coronavirus
Q202H8_CVHSA Spike glycoprotein OS=Human SARS coronavirus
Q202G5_CVHSA Spike glycoprotein OS=Human SARS coronavirus

**Table 2.** S1 proteins from SARS-CoV-2 (GISAID).

BetaCoV/Wuhan/IVDC-HB-04/2020
BetaCoV/Wuhan/IVDC-HB-01/2019
BetaCoV/Wuhan/IVDC-HB-05/2019
BetaCoV/Wuhan/IPBCAMS-WH-01/2019
BetaCoV/Wuhan/WIV04/2019
BetaCoV/Wuhan-Hu-1/2019
BetaCoV/Nonthaburi/61/2020
BetaCoV/Nonthaburi/74/2020
BetaCoV/Wuhan/WIV07/2019
BetaCoV/Wuhan/WIV06/2019
BetaCoV/Wuhan/WIV05/2019
BetaCoV/Wuhan/WIV02/2019
BetaCoV/Wuhan/HBCDC-HB-01/2019
BetaCoV/Zhejiang/WZ-01/2020

To identify the host proteins involved in the attachment and/or internalization of the SARS-CoV-2, the UniProt database ( https://www.uniprot.org) was screened by ISM for human proteins with the dominant peak on the frequency F(0.478). The list of human proteins that have a dominant peak in IS at the frequency F(0.478) are given in Table 3. According to the IS criterion, these proteins are potential candidate interactors with the SARS-CoV-2 S1 protein. Further, literature data mining was performed to identify which proteins presented in Table 3 might be involved in the processes of infection with human coronaviruses. This analysis revealed that the actin protein plays an important role in the early entry events during human coronavirus infections ^14^. Actin proteins were selected as the best candidate interactors for the SARS-CoV-2 among the host proteins that are characterized with frequency F(0.478). Figure 5c shows that CS of actins from different mammalian species ( Table 4) contains the dominant peak on F(0.478), suggesting that actin probably encodes the conserved information important for their biological function.

**Table 3.** Human proteins ( uniprot.org) with the dominant peak on the frequency F(0.478) in the informational spectrum.

ABCB8_HUMAN ATP-binding cassette sub-family B member 8, (Human)
ACTB_HUMAN Actin, cytoplasmic 1 (Human)
ACTC_HUMAN Actin, alpha cardiac muscle 1 (Human)
ACTK_HUMAN Kappa-actin (Human)
ACTS_HUMAN Actin, alpha skeletal muscle (Human)
ATL3_HUMAN ADAMTS-like protein 3 (Human)
AUP1_HUMAN Ancient ubiquitous protein 1 (Human)
CA064_HUMAN Putative uncharacterized protein C1orf64 (Human)
CETN2_HUMAN Centrin-2 (Human)
CPNE1_HUMAN Copine-1 (Human)
CR034_HUMAN Uncharacterized protein C18orf34 (Human)
CSEN_HUMAN Calsenilin (Human)
EXOC4_HUMAN Exocyst complex component 4 (Human)
F108B_HUMAN Abhydrolase domain-containing protein FAM108B1 (Human)
FGF13_HUMAN Fibroblast growth factor 13 (Human)
FRMD1_HUMAN FERM domain-containing protein 1 (Human)
GCDH_HUMAN Glutaryl-CoA dehydrogenase, mitochondrial (Human)
GKN2_HUMAN Gastrokine-2 (Human)
GPDA_HUMAN Glycerol-3-phosphate dehydrogenase [NAD+] (Human)
HPS1_HUMAN Hermansky-Pudlak syndrome 1 protein (Human)
HXK3_HUMAN Hexokinase-3 (Human)
IL23R_HUMAN Interleukin-23 receptor (Human)
KAD3_HUMAN GTP:AMP phosphotransferase mitochondrial (Human)
KRA71_HUMAN Keratin-associated protein 7-1 (Human)
LGMN_HUMAN Legumain (Human)
MYOG_HUMAN Myogenin (Human)
NTR2_HUMAN Neurotensin receptor type 2 (Human)
RD3_HUMAN Protein RD3 (Human)
S2543_HUMAN Solute carrier family 25 member 43 (Human)
SMDF_HUMAN Neuregulin-1, sensory and motor neuron-derived factor (Human)
SOX17_HUMAN Transcription factor SOX-17 (Human)
THOC4_HUMAN THO complex subunit 4 (Human)
TXND1_HUMAN Thioredoxin domain-containing protein 1 (Human)
VATE2_HUMAN Vacuolar ATP synthase subunit E 2 (Human)
ZN516_HUMAN Zinc finger protein 516 (Human)

**Table 4.** Mammalian actin proteins ( uniprot.org).

ACTB_HUMAN Actin cytoplasmic 1 OS=Homo sapiens
ACTB_MOUSE Actin cytoplasmic 1 OS=Mus musculus
ACTC_HUMAN Actin alpha cardiac muscle 1 OS=Homo sapiens
ACTB_CAVPO Actin cytoplasmic 1 OS=Cavia porcellus
ACTS_MOUSE Actin alpha skeletal muscle OS=Mus musculus
ACTB_PONAB Actin cytoplasmic 1 OS=Pongo abelii
ACTA_MOUSE Actin aortic smooth muscle OS=Mus musculus
ACTH_MOUSE Actin gamma-enteric smooth muscle OS=Mus musculus
ACTG_MOUSE Actin cytoplasmic 2 OS=Mus musculus
ACTB_MESAU Actin cytoplasmic 1 OS=Mesocricetus auratus
ACTG_HUMAN Actin cytoplasmic 2 OS=Homo sapiens
ACTS_RABIT Actin alpha skeletal muscle OS=Oryctolagus cuniculus
ACTH_HUMAN Actin gamma-enteric smooth muscle OS=Homo sapiens
ACTC_MOUSE Actin alpha cardiac muscle 1 OS=Mus musculus
ACTC_RAT Actin alpha cardiac muscle 1 OS=Rattus norvegicus
ACTA_HUMAN Actin aortic smooth muscle OS=Homo sapiens
ACTG_RAT Actin cytoplasmic 2 OS=Rattus norvegicus
ACTB_RAT Actin cytoplasmic 1 OS=Rattus norvegicus
ACTB_BOVIN Actin cytoplasmic 1 OS=Bos taurus
ACTS_HUMAN Actin alpha skeletal muscle OS=Homo sapiens

The data mining of the PubMed database ( www.ncbi.nlm.nih.gov/pubmed/) also showed that actin protein plays an important role in the rapid virus cell-to-cell spread and dissemination of infection ^15^. Additionally, the actin filament reorganization is a key step in lung inflammation induced by systemic inflammatory responses caused by infectious agents ^16^. These findings indicate that interaction between actin proteins and the S1 could be involved in the infection and pathogenesis of SARS-CoV-2. In consequence, the possibility to interfere on this interaction might represent a valid hypothesis for development of promising prevention and therapeutic strategies.

The here suggested role of actin in SARS-CoV-2 infection and pathogenesis was experimentally confirmed few months after submission of this manuscript. The ultrastructural analysis of SARS-CoV-2 interactions with the host cell via high resolution scanning electron microscopy revealed the cell-to-cell transmission of virus which is mediated by actin ^17^. The recent analysis of the SARS-CoV-2 RNA–protein interactome in infected human cells confirmed this finding ^18^. It was also demonstrated that spike protein of SARS-CoV-2 targets to the dendritic spines of neurons, which are F-actin-enriched structures [X]. This result suggests that modulation of the neural morphology by control of F-actin dynamics or rearrangement is responsible for the neurological manifestations observed in COVID-19 patients ^19^.

Interestingly, further data mining revealed that ibuprofen (FDA approved drug with excellent safety record) attenuates interleukin-1β-induced inflammation as well as actin reorganization ^20^. Actin was also found to be the primary component by which ibuprofen can bind to the tissue in different organs ^21^. This suggests that ibuprofen might impact the SARS-CoV-2-induced disease by indirect interaction with actin proteins. Previously, ibuprofen was predicted as a candidate entry inhibitor for Ebola virus using the same *in silico* approach ^22^, and this prediction was confirmed experimentally at a later time point ^23, 24^. Actin also represents the quercetin-binding protein, suggesting that this natural compound could be use in prevention and treatment of COVID-19 ^25^. The recent study performed on 113 healthcare workers working in areas with a high risk of COVID-19 demonstrated the synergic effect of quercetin and vitamin C against COVID-19 ^26^. These results prompt the possibility to experimentally test the effects of ibuprofen and quercetin on SARS-CoV-2 infection under *in vitro* and *in vivo* conditions.

*In silico* methods are considered very important tools to generate first hypotheses and identify first drug candidates against newly discovered agents, like in the case of SARS-CoV-2, especially in the short-term. ISM, a technology based on electronic biology, allowed identifying potential importance of human actin proteins for viral infection/dissemination as well as one FDA approved drug that may have an indirect antiviral activity within weeks of the initial outbreak. However, additional experiments are required to confirm our initial findings.

In conclusion, results of the presented *in silico* analysis suggest the following: (i) the newly emerging SARS-CoV-2 is highly related to SARS-CoV and, to a lesser degree, MERS-CoV, and ACE2 is a likely receptor of it; (ii) civets and poultry are potential candidates for the natural reservoir of the SARS-CoV-2, (iii) human actin proteins possibly participate in attachment/internalisation of SARS-CoV-2, (iv) drugs which interact with actin proteins (e.g. ibuprofen, quercetin) should be investigated as possible therapeutics for treatment of SARS-CoV-2 infection, and (v) domain 266-330 of S1 protein from the SARS-CoV-2 represents promising therapeutic and/or vaccine target. Further research on these issues are needed, including the development of reverse genetics and animal models to study the biology of SARS-CoV-2. Due to the fast evolving of scientific knowledge on SARS-CoV-2, the first prediction has been already confirmed, while the chicken as potential candidate as intermediate host has not been supported. Importantly, link between ibuprofen/actin interactions and viral entry remains an exciting path for future therapeutic investigations.

## Data availability

### Underlying data

Sequence data of the viruses were obtained from the GISAID EpiFlu™ Database. To access the database each individual user should complete the “ Registration Form For Individual Users”, which is available alongside detailed instructions. After submission of the Registration form, the user will receive a password. There are not any other restrictions for the access to GISAID. Conditions of access to, and use of, the GISAID EpiFlu™ Database and Data are defined by the Terms of Use.
