# Supplementary material for: Use of the informational spectrum methodology for rapid biological analysis of the novel coronavirus 2019-nCoV: prediction of potential receptor, natural reservoir, tropism and therapeutic/vaccine target
Source: F1000Res. 2021 Jan 6;9:52. Originally published 2020 Jan 27. [Version 4] doi: 10.12688/f1000research.22149.4 (PMC7202090; doi:10.12688/f1000research.22149.4)
Supplement: Peer review history

# Peer review: Reviewer response for version 4

**DOI:** 10.5256/f1000research.34828.r76710

**Published:** 2021-01-07

**Authors:** Maria Salvato

**Affiliations:** 1 Institute of Human Virology, University of Maryland, Baltimore, MD, USA; 2 STARBIOS2 (EU-funded consortium), Baltimore, USA

It is unclear which new data demonstrate that actin is an important new therapeutic and preventing target for COVID-19 disease. Is it the data referred from other authors? The cross-spectra frequency of .478 of Mammalian Actin and SARS-CoV-2 are the same, but does this vary depending on whether the actin species and the SARS-CoV host species match? In other words, does a human SARS-CoV2 have the same frequency as a bat actin? The figure legend should specify which mammalian host the "mammalian" actins represent.

Is the work clearly and accurately presented and does it cite the current literature?

Yes

If applicable, is the statistical analysis and its interpretation appropriate?

Yes

Are all the source data underlying the results available to ensure full reproducibility?

Yes

Is the study design appropriate and is the work technically sound?

Yes

Are the conclusions drawn adequately supported by the results?

Yes

Are sufficient details of methods and analysis provided to allow replication by others?

Yes

Reviewer Expertise:

Immunology, viral pathogenesis, viral genetics

I confirm that I have read this submission and believe that I have an appropriate level of expertise to confirm that it is of an acceptable scientific standard.

# Peer review: Reviewer response for version 3

**DOI:** 10.5256/f1000research.26010.r62772

**Published:** 2020-05-05

**Authors:** Maria Salvato

**Affiliations:** 1 Institute of Human Virology, University of Maryland, Baltimore, MD, USA; 2 STARBIOS2 (EU-funded consortium), Baltimore, USA

The authors have made the minor corrections requested and the manuscript looks like a valuable contribution to the literature.

Is the work clearly and accurately presented and does it cite the current literature?

Yes

If applicable, is the statistical analysis and its interpretation appropriate?

Yes

Are all the source data underlying the results available to ensure full reproducibility?

Yes

Is the study design appropriate and is the work technically sound?

Yes

Are the conclusions drawn adequately supported by the results?

Yes

Are sufficient details of methods and analysis provided to allow replication by others?

Yes

Reviewer Expertise:

Immunology, viral pathogenesis, viral genetics

I confirm that I have read this submission and believe that I have an appropriate level of expertise to confirm that it is of an acceptable scientific standard.

# Peer review: Reviewer response for version 2

**DOI:** 10.5256/f1000research.24526.r61263

**Published:** 2020-04-16

**Authors:** Maria Salvato

**Affiliations:** 1 Institute of Human Virology, University of Maryland, Baltimore, MD, USA; 2 STARBIOS2 (EU-funded consortium), Baltimore, USA

This manuscript presents a machine-learning analysis of the published sequences of the novel 2019-nCoV. The authors use the Informational Spectrum Method (ISM), a virtual spectroscopy method for protein analysis based on the electronic properties of each amino acid. Their goal is to identify sites on the virus most likely to interact with other molecules like drugs, antibodies or viral receptors. 

 Due to the rapidity of this field and the time elapsed since the manuscript was submitted (Jan 27), most of their conclusions are no longer new: nCoV is most related to SARS-CoV and less to MERS-CoV, ACE2 is a likely receptor, the natural reservoir might be civets and poultry, human actin proteins participate in internalization, ibuprofen that interacts with actin proteins should be investigated as a therapeutic, and finally that domain 266-330 of the S1 protein should be targeted by drugs or vaccines.

 It is a nice piece of work. The conclusions could be updated, for example they could say that the first of these predictions are supported by recent publications, that bat CoVs now appear to be the most closely related and bats are more likely to be the natural reservoir, and the link between ibuprofen/actin interactions and viral entry remains an exciting path for future therapeutics. 

**Minor corrections**

- In the first sentence of “Update” the authors refer to ‘these proteins’…do they mean Actin and SARS-CoV proteins? If they only mean actin they should say so, and then the sentence would read: “…actin protein is suggested as a host factor that participates in infection and pathogenesis of 2019-nCoV. Drugs modulating the biological activity of actin (e.g., ibuprofen) were suggested as candidates that should be investigated for the treatment of 2019-nCoV infection.“
- In the UPDATE, last sentences should say "...which are presented in Figure 5."
- Introduction pg 3 “Fears are mounting worldwide over the cross-border spread of the new strain of coronavirus (denoted as 2019-nCoV) originated in Wuhan,….” Instead say “…that originated in Wuhan…”
- Introduction pg 3 “…eight human 2019-nCoV isolates are available without any additional information about biological properties of the virus, beyond the morphological confirmation…” Replace “morphology” with “morphological”, it is an adjective.

Is the work clearly and accurately presented and does it cite the current literature?

Yes

If applicable, is the statistical analysis and its interpretation appropriate?

Yes

Are all the source data underlying the results available to ensure full reproducibility?

Yes

Is the study design appropriate and is the work technically sound?

Yes

Are the conclusions drawn adequately supported by the results?

Yes

Are sufficient details of methods and analysis provided to allow replication by others?

Yes

Reviewer Expertise:

Immunology, viral pathogenesis, viral genetics

I confirm that I have read this submission and believe that I have an appropriate level of expertise to confirm that it is of an acceptable scientific standard, however I have significant reservations, as outlined above.

# Peer review: Reviewer response for version 2

**DOI:** 10.5256/f1000research.24526.r60149

**Published:** 2020-03-18

**Authors:** Rita Casadio

**Affiliations:** 1 Biocomputing Group, CIG, Interdepartmental Center «Luigi Galvani» for Integrated Studies of Bioinformatics, Biophysics and Biocomplexity, University of Bologna, Bologna, Italy

Possibly the ISM method invented by the authors has high potentiality. Here, apparently, the method is applied to answer urgent questions in relation to sequence analysis of the novel coronavirus 2019-nCoV. It is interesting to verify that different papers at the moment seem to have reached the same conclusions, although in my opinion a comparison with other methods which are already published would add to the paper. On top, R.Yan *et al*, ^1^ Science 2020 4 March, beautifully detailed the putative region of the virus/ACE2 interaction. I recommend the authors quote this finding as well and if possible add the reference. This would help in validating a quite interesting method of sequence analysis.

Is the work clearly and accurately presented and does it cite the current literature?

Yes

If applicable, is the statistical analysis and its interpretation appropriate?

Partly

Are all the source data underlying the results available to ensure full reproducibility?

Yes

Is the study design appropriate and is the work technically sound?

Yes

Are the conclusions drawn adequately supported by the results?

Yes

Are sufficient details of methods and analysis provided to allow replication by others?

Partly

Reviewer Expertise:

Computational Biology, Structural bioinformatics, Functional annotation, Machine and deep learning

I confirm that I have read this submission and believe that I have an appropriate level of expertise to confirm that it is of an acceptable scientific standard.

## References

[1] : Structural basis for the recognition of the SARS-CoV-2 by full-length human ACE2. *Science*.2020; 10.1126/science.abb2762 10.1126/science.abb2762 32132184PMC7164635
